# Experiences of Using Web-Based and Mobile Technologies to Support Self-Management of Type 2 Diabetes: Qualitative Study

**DOI:** 10.2196/diabetes.9743

**Published:** 2018-05-11

**Authors:** Laura Kelly, Crispin Jenkinson, David Morley

**Affiliations:** 1 Health Services Research Unit Nuffield Department of Population Health University of Oxford Oxford United Kingdom

**Keywords:** mobile health, qualitative research, self-care, type 2 diabetes

## Abstract

**Background:**

The prevalence of type 2 diabetes is rising, placing increasing strain on health care services. Web-based and mobile technologies can be an important source of information and support for people with type 2 diabetes and may prove beneficial with respect to reducing complications due to mismanagement. To date, little research has been performed to gain an insight into people’s perspectives of using such technologies in their daily management.

**Objective:**

The purpose of this study was to understand the impact of using Web-based and mobile technologies to support the management of type 2 diabetes.

**Methods:**

In-depth interviews were conducted with 15 people with type 2 diabetes to explore experiences of using Web-based and mobile technologies to manage their diabetes. Transcripts were analyzed using the framework method.

**Results:**

Technology supported the users to maintain individualized and tailored goals when managing their health. A total of 7 themes were identified as important to participants when using technology to support self-management: (1) information, (2) understanding individual health and personal data, (3) reaching and sustaining goals, (4) minimizing disruption to daily life, (5) reassurance, (6) communicating with health care professionals, and (7) coordinated care.

**Conclusions:**

Patients need to be supported to manage their condition to improve well-being and prevent diabetes-related complications from arising. Technologies enabled the users to get an in-depth sense of how their body reacted to both lifestyle and medication factors—something that was much more difficult with the use of traditional standardized information alone. It is intended that the results of this study will inform a new questionnaire designed to assess self-management in people using Web-based and mobile technology to manage their health.

## Introduction

The number of people diagnosed with diabetes in the United Kingdom rose from 1.4 million in 1996 to nearly 3.6 million in 2016 [1-5]. By 2025, diabetes prevalence is expected to rise further to an estimated 5 million [1]. The vast majority of those diagnosed with diabetes are categorized as having type 2 diabetes (diabetes mellitus), which is estimated to cost the UK National Health Service (NHS) approximately £8.8 billion per year in direct costs and a further £13.0 billion per year in indirect costs [6]. These costs are largely spent on treating complications, such as hypoglycemia, nerve damage, heart disease, foot ulcers, and amputations, many of which can arise through the mismanagement of the condition [6]. Health services are increasingly advocating the importance of self-management to delay complications and recommend education provision and ongoing support for people with type 2 diabetes [7-9]. However, developing the knowledge and skills needed to consistently manage and perform multiple self-care tasks can be difficult to achieve, with successful management more likely in the presence of continued support [10,11]. With greater demand placed upon diabetes health services, it is increasingly difficult to support patients in the complex task of managing their diabetes. It is, however, conceivable that use of Web-based and mobile (apps and wearables) technologies could reduce strain on health care professionals and services by supporting patients in their daily decisions regarding factors, such as diet, exercise, and medication.

Many Web-based and mobile technologies have already been developed to help users manage diabetes [12-14]. The majority of these platforms offer stand-alone functions, addressing one aspect of self-management, with their use depending entirely on the individual’s desire to manage their health. For example, few mobile apps have any input from health care professionals and do not incorporate a range of basic diabetes self-care functions, such as blood glucose tracking, insulin therapy, nutrition, and physical activity [14,15]. In time, however, it is thought that technology may become more integrated with services through the transmission of data and subsequent feedback [12]. There is some evidence of this integration already in place in the United Kingdom with several systems currently being phased into existing health care pathways’ collaborative care services [16,17]. Although the effectiveness and evaluation of implementing these technologies are still underway [16], people with diabetes have indicated that the use of technology may help them to set and achieve health goals, help to track progress, access helpful information, and facilitate communication with health care professionals or peers [18]. Technology-based interventions have also been effective in reinforcing diabetes self-care behavior, although some behaviors may still be best reinforced in person [19]. Improvements, for example, have been shown in relation to being active, healthy eating, problem solving, and blood glucose control [20-24]. In contrast, the effects of technology-based interventions on behaviors related to taking medicine and coping are less clear and are not always as effective when compared with “in-person” delivery [11,19]. Research has also indicated that some mobile apps on the market carry risks to the patient, for example, incorrect insulin dosage calculations following data input error [25].

To broaden our understanding of how Web-based and mobile technologies can support self-management, further research is needed. Key biomedical outcomes, such as HbA_1c_ (glycated hemoglobin) levels, of using technologies designed to support self-management are important; however, assessing these outcomes alone neglects the complexity of how technology can assist a person to develop skills to live well with type 2 diabetes. An in-depth view on people’s perspectives of using such technologies is needed to get an insight into how they can influence daily management while taking into account broader social and contextual factors. Of the relatively small proportion of diabetes-related qualitative studies in the past 30 years, only one-quarter looked at the aspects of self-management, with an even smaller number looking specifically at the experiences of using devices to aid self-management [26]. The aim of this qualitative study was to gain an insight into the experiences and views of those using Web-based or mobile technologies to support the management of type 2 diabetes.

## Methods

### Design and Ethics

Qualitative in-depth interviews were used to explore experiences of using Web-based and mobile technologies to support the self-management of type 2 diabetes. Ethical approval for this research was granted by the Medical Sciences Inter Divisional Research Ethics Committee of the University of Oxford (reference MS-IDREC-C1-2015-109).

### Study Participants and Recruitment

Participants were aged ≥18 years with a (self-reported) clinical diagnosis of type 2 diabetes and experience of using one or more technology-based resource to support self-management. Participants were recruited through Web-based advertisements on Diabetes UK and other diabetes-related online forums. Advertisements included an electronic link that provided further information about the study and a portal to collect contact details, demographic information, and health-related technology use. The aim of this recruitment strategy was to gain a rich and comprehensive insight into experiences of using technology to support self-management among people with a range of characteristics within the study timeframe. Responders were contacted to explain the study further and arrange an in-depth interview.

As the research aim was focused, the recruitment strategy specifically targeted rich sources of data (ie, experienced users of Web-based or mobile technologies), and interviews were conducted by an experienced interviewer, it was thought that the sample size was likely to be small [27]. A specific sample size was not predetermined, however, and sampling remained continuous throughout the study until it was believed that data saturation had been achieved (ie, where no new themes are appearing) [28].

### Data Collection

In-depth interviews were conducted either face-to-face or over the telephone over a 12-week period. A topic guide was informed through relevant literature relating to self-management in people with type 2 diabetes and their use of technology-based systems. Topics in the interview guide broadly included the following: knowledge and understanding, controlling and managing symptoms using technology, self-monitoring, tailored goals, dealing with complications, use of services, and feeling supported. Prompts were used to gain a deeper understanding of participant responses on important topics. Participants were also encouraged to discuss any other topics they deemed appropriate. Interviews lasted, on average, 48 min and were recorded and transcribed. Transcription of the interviews was outsourced and accuracy checked on their return by LK. Informed consent was obtained before commencing interviews, and participants were given a £20 voucher for taking part.

### Data Analysis

Interview transcripts were analyzed using the framework method, allowing the authors to look at the data and conduct analysis in a systematic and comprehensive manner [29]. The framework method supports thematic qualitative analysis and consists of 5 stages: (1) familiarization with the interview data; (2) identification of a thematic framework to allow emerging issues, concepts, and themes to be listed; (3) indexing transcripts according to the thematic framework; (4) charting data through a process of extracting and synthesizing it to allow within-case and between-case comparison; and (5) mapping and interpretation of data [29,30]. All authors became familiarized with the transcripts and devised a suitable coding guide. Using a deductive approach, codes were preselected based on previous literature; however, analysis did allow for open coding where unexpected codes arose [31]. After the first few transcripts were independently coded by 2 authors to check for consistency, indexing of transcripts was performed using QSR International’s NVIVO software [32]. Charting of summarized data was performed in EXCEL with illustrative quotes from participants added in comment boxes. On its completion, the charting document was circulated among the team for discussion of commonalities and differences between the data and themes finalized [31].

## Results

### Characteristics

A total of 10 women and 5 men took part in the semistructured interviews. The average age was 55.4 years (SD 10.68, range 41 years). A number of additional long-term conditions were reported including sciatica, psoriasis, osteoarthritis, asthma, cancers (breast, kidney, prostate), addictions (alcohol, prescribed pain killers), chronic obstructive pulmonary disease, high blood pressure, high cholesterol, removal of pancreas, ovarian cyst, Asperger syndrome, kidney disease, and stroke. At the time of being interviewed, the time since diagnosis of type 2 diabetes ranged from 3 months to 24 years. Of the 15 participants, a total of 8 participants (53%) reported being in either full- or part-time employment. Moreover, 12 participants (80%) described themselves as white British, whereas 3 participants (20%) described themselves as white “other.”

Participants reported use of technology for the following purposes: recording and monitoring blood glucose (15/15, 100%), sourcing or logging nutritional information (11/15, 73%), accessing peer communication and support (8/15, 53%), sourcing or logging sport and exercise information (11/15, 73%), sourcing general information about diabetes (14/15, 93%), accessing their GP website (8/15, 53%), accessing personalized Web-based platforms (7/15, 47%), coping or reducing stress (2/15, 13%), and preparation for a consultation (4/15, 27%).

### Themes

#### Information

Participants felt empowered to make informed decisions with the help of instant and in-depth information found on diabetes-related websites and forums. Participants reported initiating their own online research. However, some also reported being guided by health care professionals when looking for specific information. One woman described how informed, positive changes to her diet helped her to lose weight, reduce her medication, and feel better about herself:

Well when I had my last HbA_1c_ [reading], the last two have been high, far higher than I want them to be… So I decided myself to do something about it. I read a lot, with a little bit of support from the doctor on what to get...about low carbohydrate diet and that’s where I have been using internet stuff. It’s a 10-week low carbohydrate diet... I found it through the Diabetes UK website... I have been following it and doing that, using their forum, which has all sorts of people talking on it. If you have a question you can ask and the people give advice... what I have found, because I have kept the carbohydrates low since the middle of January–we’ve dropped two of my pills...I have actually felt sparkier, brighter and the added benefit of all of that is that I have lost 8-9 lbs.

Participant 8, female, 64 years

Online information also made participants aware of the potential risks and side effects of decisions they made when managing their health, particularly in relation to medication. One woman, who had experienced significant hair loss and depression after being prescribed medication in the past, used the Internet to research subsequent medications prescribed:

...when it [medication] was prescribed to me I went onto the net to find out more about it. It doesn’t always say everything in the leaflet that you have and I am aware that with [medication] it was tested on lab rats and lab mice. And it can cause thyroid cancer, I am aware of that. What I do find, not distressing, annoying really—what I find astonishing is that when it was prescribed at no time was I ever told that. I know that because I went and I did my research to find that out because I wanted to know what it was that I was taking. I know that for every action there is a reaction, so to me I looked at it that this action meant there was a reaction somewhere... I would have [preferred to know the risks], because then it’s my choice.

Participant 5, female, 52 years

#### Understanding Individual Health and Personal Data

Participants sought to understand the impact of diet and, in some cases, exercise on their blood glucose levels. All participants took regular blood glucose readings. In most cases, readings were tracked and monitored throughout the day, with 2 participants wearing a continuous blood glucose (CBG) monitor that offered readings (and alerts) in real-time. Regular monitoring enabled participants to learn about daily patterns in their blood glucose levels and gave a greater understanding of its relationship with diet and exercise. Having a greater understanding of how their body behaved contributed to an overall sense control when managing diabetes:

I don’t measure my bloods all day every day, but I do it as a testing thing to basically learn from it. I’ve found from it I know which foods I can eat and which has less effect and I’ve discovered that exercise after a meal makes a really big difference for bringing down the blood sugar...to be honest I think...that’s possibly been one of the most useful tools that I’ve had.

Participant 2, female, 55 years

Technology was particularly useful when making sense of large amounts of data, especially if an app tracked more than one aspect of health (eg, blood glucose readings plus physical activity plus diet). One man describes how graphs helped present large amounts of longitudinal data to give an overall picture of health:

The app on my phone has a graph and how many steps is your target for walking in a day, how much water intake and what you are eating so you can record that. So it’s easy to trace that way. If you see the paper you can see the spike... It records manually your blood sugar levels and it has a little graph on the screen...then you can see over a couple of weeks how you are doing.

Participant 4, male, 60 years

#### Reaching and Sustaining Goals

Having an in-depth understanding of the relationship between factors such as diet, exercise, and medication on blood glucose levels helped participants to refine their goals when managing diabetes. Looking for patterns in personal data helped participants to consider adjustments to their lifestyle that they could maintain:

[Since using the Libre sensor]... I’ve actually managed to get my average [blood glucose level] down from 7.8 to 6.8 [mmol/L]... Because I can see more patterns of what’s going on, I’m knocking it down...It gives me a little bit more control into my diet... I can see sometimes I have something to eat and it will peak quite high and I think what the hell have I eaten that’s peaked so high... And I sort of think what can I do to change that?...in the mornings I was having fruit on top of my cereal... You know it was only about a tablespoon of fruit, and then it was really peaking quite a lot. I thought rather than do that, I will have the fruit later on in the day when I am dropping down a bit, which has worked for me.

Participant 21, female, 54 years

Technology not only enabled participants to investigate deviations in blood glucose levels from their targeted range, it could also act as a warning system to alert participants to make them more aware of when they needed to be careful about specific aspects their management for the remainder of the day:

The thing that I really like about [app] is that I can log everything that I eat—it gives me a daily diary. It works out my calorie level, it works out my sugar levels and I can log everything I have. So I can keep a track on my fats, I can keep track of my carbohydrates and, more importantly, I can keep track of my sugar, so I know, if it tells me the sugar—right you’ve got to behave this evening, or I can’t have this, this evening. And I truly wouldn’t be without it, it’s a real informative app, you know.

Participant 5, female, 52 years

A total of 7 participants (7/15, 47%) discussed how apps had helped to remind and motivate them to engage in healthy self-care behaviors. Real-time logging and in-app alert systems, such as the CBG sensors and activity trackers, were particularly useful for warning participants to take action according to their predefined goals before it was too late:

[Apple watch] reminds me when I am not doing everything I should do... It’s got my activity targets in it... if I haven’t walked enough I know from the tracker that I haven’t done the required number of steps ... It’s also set to remind me that I need to get up from my desk and walk around every so often. So if it reminds me somewhere where it’s convenient for me to get up and do anything about it, I get up and do something about it.

Participant 18, female, 58 years

In addition to progress reports and practical reminders to reach targets, technology emotionally supported participants by allowing them to record feelings or feel encouraged to continue practicing good behaviors through feedback. Participants described how seeing progress and receiving positive signals motivated them to continue managing their health. A small number of participants had experience of using mobile apps that used gamification techniques to encourage motivation. Although one man felt this style of encouragement was not pitched to his level, one woman described how these techniques kept her engaged and motivated:

[The app] is quite user friendly, it’s got this kind of image...you put your [blood glucose level] figure in to control your blood sugar monster...it’s quite fun as well, so I think it’s motivational... When you put your blood sugar in, the more that you do, the more you kind of use those stickers to get your blood sugar and this little kind of monster gets chained up, he is literally put in chains. So the motivation is to chain up the monster.

Participant 14, female, 44 years

#### Minimizing Disruption to Daily Life

Technology was used as a tool to maximize convenience when managing diabetes and to minimize the chances of disruption to everyday life. CBG sensors reduced the time taken to manually check blood glucose levels and conveniently gave alerts, warning the wearer when they were approaching specific thresholds. This resulted in minimum disruption to daily plans. For example, wearers could schedule appointments or get to and from work knowing their blood glucose levels would be within the “safe limits” for driving:

I like the alarm [on the CBG monitor], I often have it off at work, not because I am at work, just because my blood sugar is usually higher at work. And I can look at it if I want to see it. But then in the evening, which is, when I have more hypos, or I am out doing whatever I am doing, I have the alarms on then. It says things like you are low and then you have to wait 45 minutes before you can drive... So that never really happens with the alarms because if I have to drive from work, I set the alarm at 5 [mmol/L] and then I never get that low blood sugar, so I am going to have to wait to drive. You know it doesn’t get in the way... Whereas before, often I would go low in the evening, because I often do walking the dog and then if I had one [hypo] in the evening I had to wait an hour and I would be an hour late.

Participant 15, female, 29 years

Apps were used as a management aid when participants were not in their usual routine. Participants discussed using a range of apps that helped them research the nutritional content of food on a menu when dining out. This resource reduced the anxiety associated with not knowing what to eat in a restaurant. Other useful feature of apps when eating out was the opportunity to log food on the go so that diet could be accurately recorded with minimal effort:

So I went out to lunch with a friend yesterday lunchtime and I sat there with my phone, worked out how much carbs I had got in my meal and just sat there at the table and just tapped it in. I tapped in how much insulin I was taking, instead of thinking “Right I must remember to do that when I get home.” And then by the time you get home, things happen and you have forgotten it. And that has been brilliant. Also because it is so easy to do, because it is there, you do it... My husband said it has helped with my discipline, it really has.

Participant 12, female, 70 years

Although technology was viewed as a largely helpful and convenient resource, some frustrations were reported regarding ease of use. Inconveniences included difficulties in entering data (eg, apps requiring the use of imperial units instead of metric units) or the requirement of purchasing new equipment to wirelessly input data to an app:

There is nothing wrong with the app itself, but it was designed to be used with an iHealth BP monitor. I have a glucose monitor and I thought I am not spending another couple of hundred quid, the monitors I have got are what my doctor wanted me to have and to use them...[to input readings], manually it was too much like hard work. I just wanted to open it up, put the figures in and shut it down. So that didn’t get very far.

Participant 13, male, 70 years

#### Reassurance

Participants reported feeling reassured when they knew they were managing their diabetes well. In 6 cases, where blood glucose levels were consistently stable, participants described only taking blood glucose readings periodically, acting as a “checking” system, whereas another participant described checking her blood glucose levels periodically to reassure herself that symptoms she was experiencing were not related to her diabetes:

Sometimes the feeling of being anxious is the same as being hyper. So I wanted to be sure am I nervous or am I low.

Participant 15, female, 29 years

Information found online also offered reassurance to participants. Forums, in particular, were useful for instances where participants did not fit the text book patient and were looking for nonstandard advice from peers:

Health services are wonderful at the norm; they are not that good when you don’t follow what the textbook says should be happening. And that’s where the forums can come in, because actually it’s quite reassuring there is an awful lot of non-standard people out there.

Participant 18, female, 58 years

#### Communicating with Health Care Professionals

Technology used in daily management routines, usually apps, aided participants when describing their general health trends and communicating with health care professionals during consultations. A total of 7 participants (7/15, 47%) cited times where their electronic recordings helped them during consultations by providing real-time “evidence” on daily patterns. Providing a detailed picture of their daily well-being conveyed whether their current management practices were working effectively for them and enabled them to have some control of how to proceed with their care. Moreover, 3 participants (3/15, 20%) reported a change in treatment due to the use of technology within their consultation. One man gives an account of how technology helps him to be a partner in his health care decisions:

When I am talking to the doctor I don’t want to be sort of trying to remember what happened six weeks ago... We’ve both got a very good link here, a nice graph that she can look at as a doctor and say—“What happened there?”—“Yes, this one is working out quite well”... I can also share it with her; I can export it to her... I can just say whether I want one day, month, or year or I can do a custom layout and I can send it as a PDF, CSV or HTML... Sometimes my doctor says, “Any chance of sending a chart a few days before you come in?,” so they can look at it and I say—“Yes fine, off you go”... my view is if you have got 10 minutes, let’s make it really count. So I don’t like going in and spending five minutes explaining what my readings have been.

Participant 13, male, 70 years

Although this approach generally helped to make efficient use of consultation time, one respondent noted the presentation of data could be misleading in consultations as her app used thresholds to color code blood glucose reading ranges. In a busy consultation, this could be misinterpreted:

...it’s very visual and you can go through it and you can compare one week’s total to another week...my GP will look at the app...It comes up in different colours, so they generally look at the colours literally, because green is within the bounds that are acceptable and the reds are either side, whether they are too low or too high. I’ve had my GP say to me, “Oh you’re too low here” and I have pointed out it’s 3.9 and I’m normally 4.2, so I am not worried about 3.9... The monitor [readings are] about 20% either way [of true reading] I think, it’s not perfect.

Participant 14, female, 44 years

#### Coordinated Care

Electronic tools were rarely provided by or integrated with health care services. One participant, however, discussed using an electronic record that detailed medical history and upcoming appointments. This enabled him to coordinate aspects of his care. The effect was a more streamlined approach to managing his health, more certainty on what to expect when meeting health care professionals and sense of partnership in decisions:

I am a happy bunny. It’s [electronic patient record] very good; it’s got my medical history on here, linked to the NHS records direct...[It’s] Brilliant because I find in the self-management side of things, my major problem isn’t the doctor I face, it isn’t the specialists at the hospital ...my main problem is stuff getting lost between them on the admin side... So if I am seeing a doctor tomorrow and she says “I want you to do bla bla bla,” I will get an appointment for you within three weeks, I can check my records and make sure it has been done and if it hasn’t been done, it helps me ring up and chase it... I am a strong believer in knowledge is power basically... I enjoy being an equal partner.

Participant 13, male, 70 years

## Discussion

### Principal Findings

Health care services are under increasing pressure to cope with the rising number of people diagnosed with type 2 diabetes. Patients need to be supported to manage their condition to improve well-being and prevent further diabetes-related complications arising. Web-based and mobile technologies may offer one solution to supporting those with type 2 diabetes with this management, but there is currently limited qualitative research into their effect on self-management [26]. This study explored the views and experiences of men and women using technology to support self-management.

This research demonstrates how technology supported users to maintain individualized and tailored plans when managing their health. It was clear that participants wanted to feel informed when managing their health, feel reassured that they were managing their health effectively, and, where possible, wanted self-care tasks to be minimally intrusive to their daily life. Technologies enabled users to get an in-depth sense of how their body reacted to both lifestyle and medication factors, something that was much more difficult with the use of traditional “standardized” information alone. Understanding how their body reacted to lifestyle and medication factors was welcomed among the sample and this understanding was enhanced through resources which helped to organize and make sense of vast amounts of longitudinal data (eg, through output in the form of graphs and charts). Wearable devices, such as the CBG monitors, were particularly helpful in that they offered real-time solutions and alerts. The use of available technologies appeared to motivate participants to achieve and sustain healthy goals when managing their diabetes. It is important to note, however, that participants responded to aspects of technology designed to motivate and incentivize engagement in different ways. The success of engagement features, which Nelson et al [33] refer to as functions for “engagement promotion,” was dependent on the preferences of the user. For example, although one woman enjoyed gamification techniques (unlocking achievements), one man considered them as childish elements of an otherwise useful resource. Participants also voiced frustrations with the need to buy new, compatible devices (such as wireless devices with Bluetooth functionality) and difficulties in entering data (eg, the requirement of inserting imperial units).

Participants were very focused in their wants and needs from technology and indicated that they “shopped around” to find mobile apps or other tools, such as CBG sensors, that targeted their requirements. If a particular app, for example, did not provide the right information or was not easy to use, they proceeded to try alternatives reasonably quickly. Furthermore, although data were not collected on how long participants had been using specific technologies, some participants did reflect on periods of high and low technology usage. For those with varying usage intensity, periods of high intensity tended to reflect an “event” (eg, the introduction of a new diet) and low usage reflected periods when they felt their blood glucose levels were under control. Understanding more about the reasons behind swift dropout when using a new mobile app and reasons for varying intensity of usage are interesting as digital health interventions are frequently faced with challenges of high attrition rates [34,35]. Attrition and periods of low usage are problematic from a research perspective when trying to prove the effectiveness of a Web-based or mobile technology; however, this study indicates that this challenge is reflective of how people use technology in real life. That is, they often use multiple apps to cater for a complex condition when self-managing, they are selective when looking for self-management tools and swiftly discard those that do not complement their lifestyle, and the intensity of their use will vary over time.

This study builds on existing research by providing an in-depth overview on how people can use multiple Web-based and mobile technologies to support diabetes management in their daily lives. Many previous studies have evaluated how a specific intervention was used [36-38]; however, this study encouraged participants to share experiences of using a combination of resources, more comparable to practices in daily life. Although the use of multiple apps may, in part, be due to the limited functions provided by one mobile app alone [12,39], this research indicated users appreciate multiple functions to have a holistic view of the relationships between various lifestyle factors on their health. Supporting previous research [40], these interviews showed that health technologies can give people with diabetes a heightened awareness of lifestyle factors on their blood glucose levels and encourage problem-solving through making changes to their diet and/or activity levels after identifying reasons for highs and lows. As this study explored current and past experiences of technologies, it also provided examples of how participants sustained changes in behavior. Related research in the context of other long-term conditions [41] can also be drawn upon to support these findings, which demonstrate the importance of having the flexibility to tailor, personalize, and prioritize self-management approaches using health technologies.

### Limitations

Some limitations of this study must be acknowledged. These are largely related to the transferability of the findings. Despite efforts to include a range of backgrounds within the sample, those from a nonwhite background were not represented and only 3 classified themselves in a non-British category. Older people, however, were represented in the sample, which was particularly welcomed given that they can have problems engaging with technology [33,42]. This sample also represented a self-selecting group of people with type 2 diabetes who were comfortable using technology. As such, these findings are limited in their transferability to people with type 2 diabetes who do not use technology and have low motivation to use technology in the management of their health. However, the authors purposely chose to recruit rich sources of data in the time available to them. Exploring possible reasons why people do not use technology in the management of their health was not in the scope of this study.

In contrast to the research reported by Ancker [43], participants in this sample were largely enthusiastic when tracking and monitoring their own health. This may have been due to self-selection; however, it may have also been related to participants being able to move on from apps that were inconvenient in favor of technology more suited to their preferences. Furthermore, although participants in this sample reported multiple long-term conditions, Ancker’s focus was on people with multiple conditions, which may have resulted in feelings of being overwhelmed. Although participants discussed negative aspects of using technology in functional terms, for example, frustration with data entry, there was limited information on possible negative health effects of using these technologies. This may, in part, be due to participants swiftly moving on from technologies that did not meet their requirements. Negative aspects of using technology to support self-management may be something that would benefit from further research in the future.

### Conclusions

Patients need to be supported to manage their condition to improve well-being and to prevent diabetes-related health complications arising. Technologies enabled users to get an in-depth sense of how their body reacted to both lifestyle and medication factors, something that was much more difficult with the use of traditional “standardized” information alone. Health care professionals who are responsible for educating and supporting those with type 2 diabetes may find Web-based and mobile technologies to be invaluable tools for engaging with their patients and tailoring information during a consultation.

These findings demonstrate how those engaged in technology use multiple apps to optimize self-management. Developers in Web-based and mobile technologies should aim to help the user manage a range of self-care tasks from one app to offer a more holistic experience. This study also highlights the difficulties for developing and assessing digital behavioral interventions due to users’ adoption of multiple technologies and swift dropout. Researchers and Web developers should place more emphasis on rates of retention in the use of technology-based interventions as opposed to rates of initial adoption of the intervention.

## Abbreviations

CBG: continuous blood glucose
HbA_1c_: glycated hemoglobin
NHS: National Health Service
